# Three-dimensional optical holography using a plasmonic metasurface

**DOI:** 10.1038/ncomms3808

**Published:** 2013-11-15

**Authors:** Lingling Huang, Xianzhong Chen, Holger Mühlenbernd, Hao Zhang, Shumei Chen, Benfeng Bai, Qiaofeng Tan, Guofan Jin, Kok-Wai Cheah, Cheng-Wei Qiu, Jensen Li, Thomas Zentgraf, Shuang Zhang

**Affiliations:** 1 School of Physics and Astronomy, University of Birmingham, Birmingham B15 2TT, UK; 2 State Key Laboratory of Precision Measurement Technology and Instruments, Department of Precision Instrument, Tsinghua University, Beijing 100084, China; 3 Department of Physics, University of Paderborn, Warburger Straße 100, D-33098 Paderborn, Germany; 4 Department of Physics, Hong Kong Baptist University, Hong Kong, China; 5 Department of Electrical and Computer Engineering, National University of Singapore, 4 Engineering Drive 3, Singapore 117576, Singapore; 6 These authors contributed equally to this work

## Abstract

Benefitting from the flexibility in engineering their optical response, metamaterials have been used to achieve control over the propagation of light to an unprecedented level, leading to highly unconventional and versatile optical functionalities compared with their natural counterparts. Recently, the emerging field of metasurfaces, which consist of a monolayer of photonic artificial atoms, has offered attractive functionalities for shaping wave fronts of light by introducing an abrupt interfacial phase discontinuity. Here we realize three-dimensional holography by using metasurfaces made of subwavelength metallic nanorods with spatially varying orientations. The phase discontinuity takes place when the helicity of incident circularly polarized light is reversed. As the phase can be continuously controlled in each subwavelength unit cell by the rod orientation, metasurfaces represent a new route towards high-resolution on-axis three-dimensional holograms with a wide field of view. In addition, the undesired effect of multiple diffraction orders usually accompanying holography is eliminated.

## Abstract (web summary)

Holographic techniques allow for the construction of 3D images by controlling the wave front of light beams. Huang *et al.* develop ultrathin plasmonic metasurfaces to provide 3D optical holographic image reconstruction in the visible and near-infrared regions for circularly polarized light.

## Graphical abstract

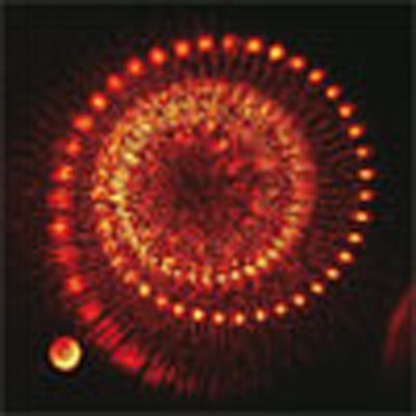

Metamaterials represent a non-traditional approach to manipulating local and far field light behaviour, leading to exotic optical phenomena such as negative refraction^1^,^2^, superimaging^3^,^4^,^5^,^6^ and invisibility cloaking^7^,^8^,^9^,^10^,^11^,^12^,^13^,^14^. The building blocks of metamaterials are function-driven artificial meta-atoms, resulting in great flexibility in tuning electromagnetic properties such as the effective electric permittivity and the magnetic permeability. Metasurfaces, a new class of metamaterials that consist of only a monolayer of planar metallic structures, have shown great promise for achieving full control of the wave front of light with low fabrication cost as they do not require complicated three-dimensional (3D) nanofabrication techniques^15^,^16^. Metasurfaces are capable of generating abrupt interfacial phase changes, and provide a unique way of controlling the local wave front at the subwavelength scale^17^,^18^,^19^,^20^,^21^,^22^,^23^,^24^,^25^,^26^,^27^,^28^,^29^,^30^. A plethora of applications have already been proposed and demonstrated by using metasurfaces such as wave plates for generating vortex beams^17^,^19^, ultrathin metalenses^22^,^23^, aberration-free quarter-wave plates (QWPs)^21^, the spin-hall effect of light^27^,^30^, polarization-dependent unidirectional surface plasmon-polariton excitation^28^,^29^,^30^ and spin-controlled photonics^30^.

Shaping the phase distribution of a wave is very important for reconstructing 3D images, a technique that has been known as holography for several decades. A hologram contains the complete information of the object beam, in contrast to photography or multiview parallax techniques, which store only information for at most a limited number of viewing directions^31^. The holographic recording itself is not an image; rather it consists of seemingly random patterns of varying intensity or phase. The hologram can be generated either by interference of a reference beam with the scattered beam from a real object or by using numerical computation to calculate the phase information of the wave at the hologram interface and encoding the phase information into surface structures by lithography, or a spatial light modulator (SLM). The latter method is usually referred to as computer-generated holography (CGH)^32^,^33^.

Recently, holography-based techniques for controlling the amplitude and phase of free-space beams have been used to achieve surface plasmon holographic displays^34^,^35^, beam shaping^36^, data storage^37^, digital holographic microscopy^38^,^39^, optical trapping and micromanipulation in atom traps or diffractive laser tweezers^40^,^41^. Two-dimensional (2D) holography, or projection, has also been experimentally demonstrated using metamaterials^42^,^43^,^44^,^45^,^46^,^47^,^48^. However, none of these techniques have achieved 3D CGH image reconstruction in the visible range, although the essence of holography lies in its capability to display 3D images. Here we demonstrate 3D CGH image reconstruction by using an ultrathin plasmonic metasurface consisting of an array of subwavelength plasmonic antennas with carefully defined orientations. The realization of the hologram by such metasurfaces is very simple and elegant because of the extremely simple relationship for encoding the phase information into the configuration of the structures, that is, the orientation angle of the nanorods. This particular feature of our metasurface is highly desired in holography as it is robust against fabrication tolerances and variation of metal properties because of the much simpler structure geometry and the geometric nature of the phase. More importantly, because of subwavelength control of the spatial phase profile, metasurfaces provide a solution for increasing the angular range of perspective for digital holograms and enhancing the space-bandwidth product of holographic systems.

## Results

### Design of metasurface hologram

For our demonstration, we utilize the abrupt phase change that occurs for circularly polarized (CP) light when converted to its opposite helicity^19^,^49^. The phase shift at the interface, ranging from 0 to 2*π*, is realized by a metasurface consisting of an array of plasmonic dipole antennas with subwavelength separation. The local phase of light transmitted through the metasurface is geometrical and solely controlled by the orientation angle *ϕ* of the individual dipole antennas as Ф=±2*ϕ*, with the sign determined by the particular combination of incident/transmitted polarization, that is, ‘+’ sign for RCP/LCP and ‘−’ sign for LCP/RCP (RCP: right-handed circular polarization; LCP: left-handed circular polarization). Importantly, the scattering amplitudes of the antennas for converting the incident light to its opposite helicity are uniform. This greatly eases the encoding procedure of phase-only holograms; therefore, circular polarization-based metasurfaces that consist of simple plasmonic nanorods can be used to record the hologram without an extra look-up table.

By using a CGH algorithm^50^, the 3D object is approximated as a collection of point sources and both the recording and image reconstruction procedures are achieved without the need for a reference beam. Further, the objects to be reconstructed are designed to have rough surfaces that give rise to diffuse reflection. As such, the amplitude information of the hologram can be entirely eliminated without decreasing the image quality. Each pixel of the hologram only contains a single subwavelength plasmonic nanorod whose orientation encodes the desired continuous local phase profile for CP light illumination. Because of the subwavelength pixel pitch, the zero-order on-axis 3D reconstruction can potentially achieve very high resolution and wide field of view (FOV). Moreover, the dispersionless nature of the circular polarization-based metasurface allows the hologram to be reconstructed at a broad range of wavelengths.

**Figure 1:**
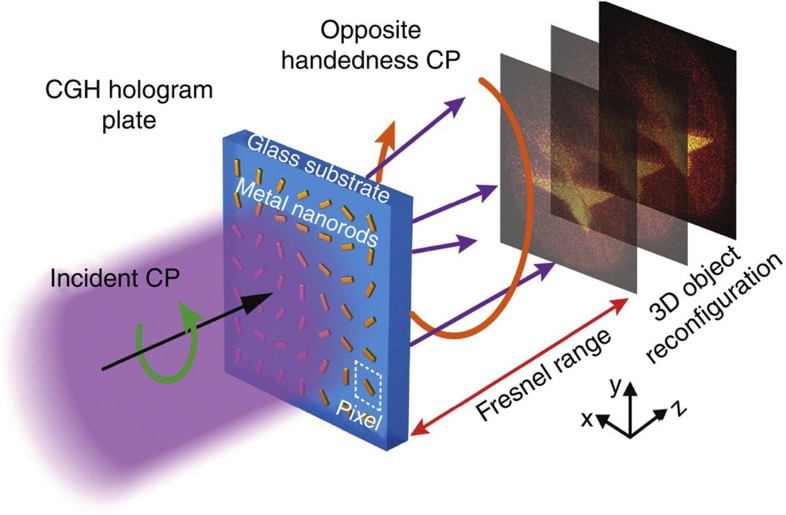
Hologram structure and reconstruction procedure. Each nanorod has the role of a pixel of diffractive element, which can generate the required continuous local phase profile with normal incidence of circularly polarized (CP) light, and only opposite handedness CP light are collected. The reconfigured 3D models are designed to appear within the Fresnel range.

Figure 1 illustrates the hologram structure and the reconstruction procedure of the 3D image. When the pixelated nanorod pattern is illuminated with CP light, it generates the desired continuous local phase profile for the transmitted light with opposite handedness. The number of pixels on the hologram is determined by the conservation of the space-bandwidth product and the sampling law, as to provide sufficient CGH object information and avoid aliasing effects. The holographic 3D image appears in the Fresnel range of the hologram. Note that if the polarization of the incident and transmitted beams are both reversed, the sign of the phase acquired is flipped, which would result in a mirrored holographic image on the other side of the metasurface.

The procedure of designing and displaying 3D holograms consists of the following steps: digital synthesis and numerical calculation of the 3D CGH, encoding the phase information into the pixelated nanorods by their orientation and reconstruction of the image by a conventional optical transmission scheme. In general, the 3D objects are set up by 3D computer-graphics software or by 3D mathematical functions in MATLAB. By using a point source algorithm, the calculated 3D objects are approximated as a collection of discrete point sources. The complex amplitude *H*(*x*, *y*) on the hologram plane can then be calculated by superimposing the optical wave fronts of all the point sources. With the proper incorporation of a Gaussian-distributed random phase for each point source, which emulates diffuse reflection of the object surface, a uniform amplitude distribution can be obtained in the hologram plane (Methods), and thus good quality holographic reconstruction with only the phase information can be achieved.

### Characterization of metasurface hologram

**Figure 2:**
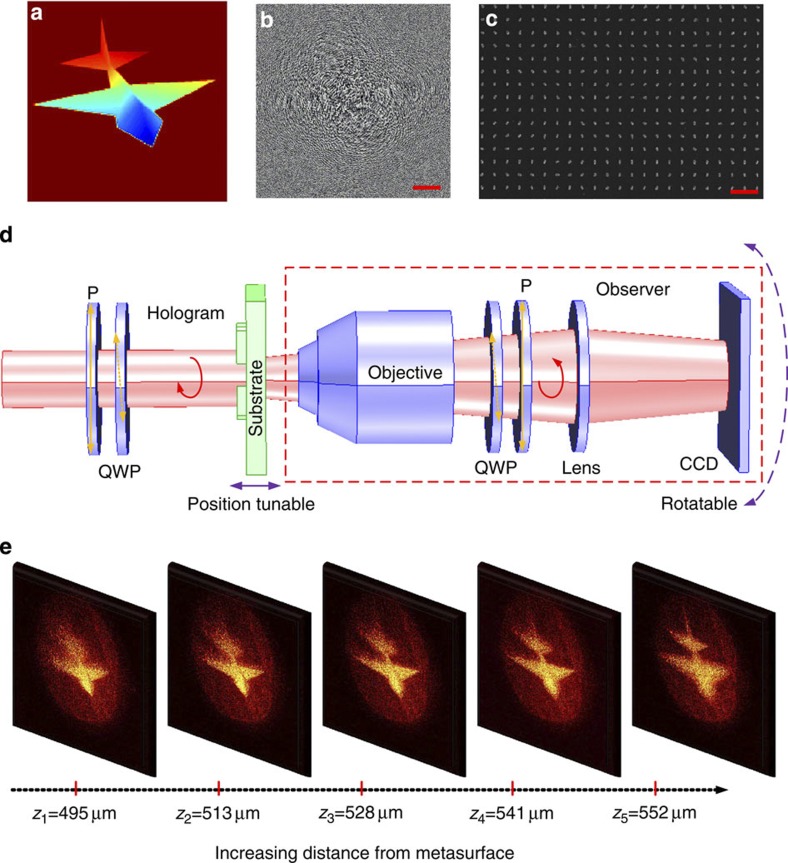
Experimental investigation of 3D holography by the metasurface. (**a**) The 3D model for a jet generated in a graphics programme. The size of the jet from left wing to right wing is 330 μm, and from head to tail is 232 μm. The dimension along the *z* direction is 48.2 μm. (**b**) Calculated hologram of the jet. Scale bar, 20 μm. (**c**) Scanning electron microscopy image of a part of the corresponding metasurface. Scale bar, 1 μm. The gold nanorods in the hologram plate are ~150 nm long and ~75 nm wide, and the pixel size *s* between two neighbouring rods along the *x* and the *y* direction is 500 nm. The entire hologram is made of 800 × 800 pixels. (**d**) Optical set-up for observation of holographic image with tuneable focus positions and rotational angular perspective. (**e**) Evolution of the appearance of jets for different focusing positions along the *z* direction with RCP illumination.

For the experimental proof of the concept, we fabricate various hologram samples, consisting of metallic nanorods, by electron beam lithography followed by deposition of 40 nm gold and a lift-off process. We first carry out the 3D holography of a solid jet model (Fig. 2a). The target 3D solid jet model is created by computer-graphics software. In the next step, the metasurface hologram for the jet pattern is designed for a wavelength of 810 nm. The target object is chosen to be submillimetre size, which is comparable to that of the hologram sample, with a sampling of 200 × 200 pixels. The hologram contains 800 × 800 pixels, with a lattice constant of 500 nm and nanorods of dimension 150 × 75 nm^2^ (Fig. 2b,c).

To determine the performance of the hologram, we use an experimental set-up as shown in Fig. 2d. A linear polarizer (P) and a QWP are positioned in front of the sample to prepare the desired circular polarization state for the illumination. Because of the submillimetre size of the reconstructed image, a × 10 (numerical aperture=0.3) magnifying microscope objective lens, in combination with another convex lens are positioned in front of the charge-coupled device (CCD) to capture the images. A second linear polarizer and QWP pair is inserted between the two imaging lenses to ensure that only light with opposite handedness is collected. To measure different viewing angles, all of the observational optical components, that is, components placed after the sample, are arranged on a rotation stage centred on the sample. Because of the finite depth of focus of the objective lens, a 3D image cannot be directly captured by the CCD imaging system. Instead, the depth information of the 3D constructed image can be analysed by gradually tuning the distance between the sample and objective. In addition, the perspective information of the 3D holographic image can be further investigated by rotating the imaging system centred at the sample. Thus, with a series of 2D images, depth and angular perspective can be demonstrated separately, allowing the 3D nature of the holographic images to be verified.

**Figure 3:**
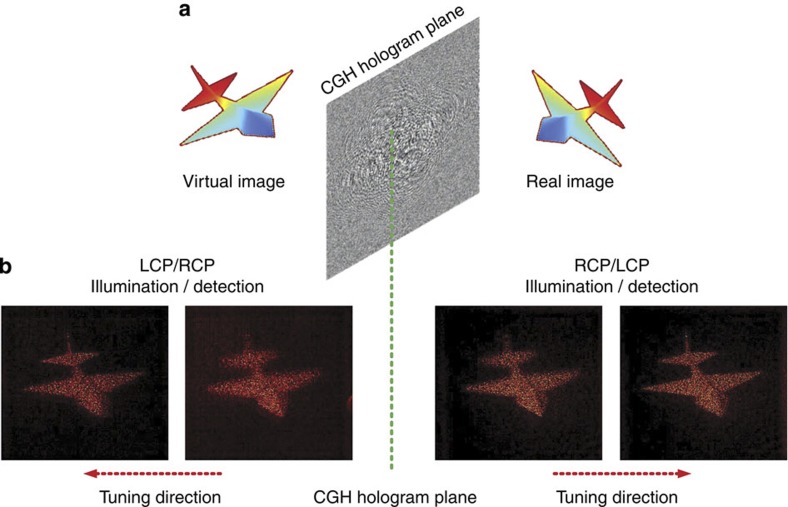
Demonstration of the real and the virtual holographic images. (**a**) Schematic illustration for the appearance of the real and the virtual holographic images of jet on both sides of the metasurface plane. (**b**) Experimentally obtained images. The real image appears on the transmission side when illuminated and detected by RCP/LCP combination, whereas the virtual image is on the opposite side when both illumination and detection polarizations are reversed. For both real and virtual images, the location where the head of the jet gives a sharp image and is therefore in the imaging focus plane is closer to the metasurface than where the tail looks clear. This verifies that the virtual image and real image are symmetric about the metasurface. The wavelength at which the images were taken is 820 nm.

Results for the holographic images of the solid jet model at different object planes are shown in Fig. 2e. At first, the imaging system is deliberately designed to have a small depth of focus. This helps to verify the 3D nature of the holographic image, as the sharp focus allows certain parts of the full 3D holographic image to be viewed selectively. In the first polarization configuration, RCP light of 810 nm wavelength is normally incident onto the sample and LCP light is detected. A real holographic image of the jet appears on the transmission side of the metasurface, as shown by the evolution of the 2D images at different distances relative to the metasurface. For *z*_1_=495 μm away from the metasurface, only the head of the jet forms a sharp image on the CCD camera, whereas the tail is slightly blurred. At a different distance of *z*_2_=552 μm, the tail now forms a sharp image on the CCD camera and the head appears blurred. The difference between *z*_1_ and *z*_2_ agrees reasonably well with the depth of the target 3D holographic object along the *z* direction, which is 48.2 μm. The phase profile of the circular polarization-based metasurface relies on the selection of different circular input/detected polarization^19^. When both of them are reversed, the phase profile is expected to be reversed as well. This indicates that there exists a virtual holographic image at the opposite side of the sample when simultaneously switching the polarizations of the illuminated and transmitted light, as schematically shown in Fig. 3a. This effect is experimentally verified by observing an image of the jet on the opposite side of the sample space (Fig. 3b). The measurement further shows that the virtual holographic image is exactly symmetric about the position of the metasurface relative to the real holographic image detected previously. That is to say, by tuning the object plane away from the hologram (metasurface), we first obtain a clear image of the jet’s head and then gradually the sharp focus shifted to the tail for both real and virtual holographic images.

**Figure 4:**
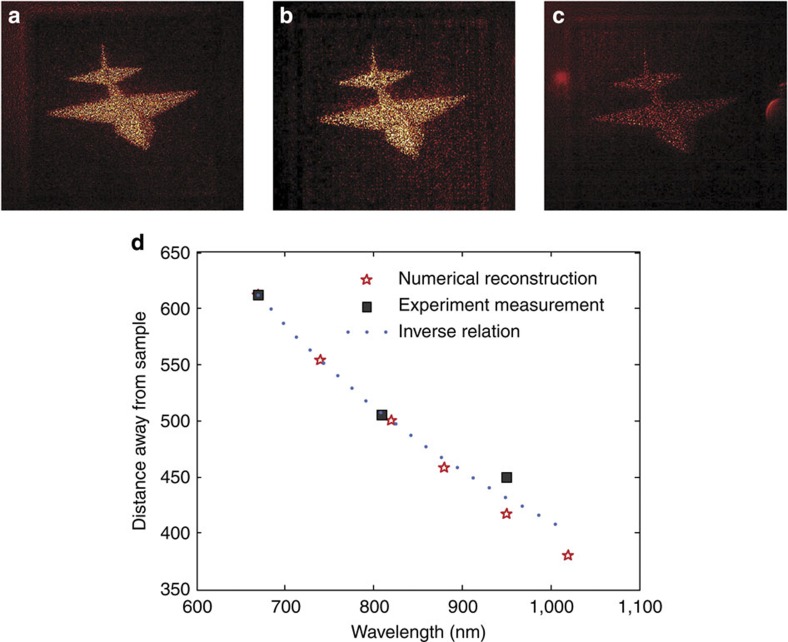
Holographic image reconstruction for different wavelengths. Recorded images for wavelengths of (**a**) *λ*_1_=670 nm, (**b**) *λ*_2_=810 nm and (**c**) *λ*_3_=950 nm. (**d**) Distance of the reconstructed holographic image from the sample surface. The red stars denote the value calculated from angular spectrum method, the square dots represent the experimental measurement and the blue dotted curve is calculated from the simple inverse relationship between the wavelength and *z* position.

The metasurface design in our experiment exhibits a dispersionless phase profile, which results from the geometric berry phase^19^. Consequently, it is expected that holograms based on such metasurfaces should be broadband, although the hologram was designed for a specific wavelength (810 nm). Figure 4a–c shows the holograms at three different wavelengths: 670, 810 and 950 nm, respectively. Note that the imaging system is now slightly modified such that the depth of focus is increased. Consequently, all parts of the jet, from head to tail, now appear sharp on the CCD camera simultaneously. The reconstructed images at these three wavelengths are almost identical; only the distance to the metasurface is varied from 620 μm at *λ*=670 nm wavelength to 450 μm at *λ=*950 nm. The change of location can be explained by the change in phase accumulated during the wave propagation due to the change in wave vector. Mathematically, the locations of the holographic images corresponding to two different wavelengths are related by:


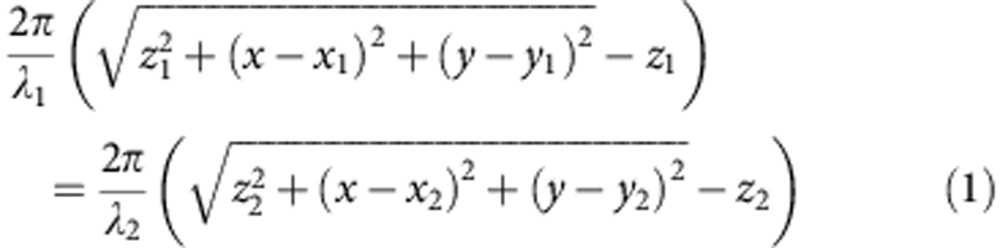


where (*x*, *y*) are the coordinates on the metasurface and (*x*_i_, *y*_i_, *z*_i_) represent the location of a particular point in the 3D holographic image at wavelength *λ*_i_. It is easy to verify that, under the paraxial approximation, the relation can be simply expressed as *x*_1_=*x*_2_, *y*_1_=*y*_2_ and *λ*_1_*z*_1_=*λ*_2_*z*_2_. Thus, the distance between the holographic image and the metasurface is approximately inverse proportional to the wavelength. This simple relationship explains very well the experimental observation as well as a more rigorous calculations of the image positions by using the angular spectrum method (Fig. 4d).

**Figure 5:**
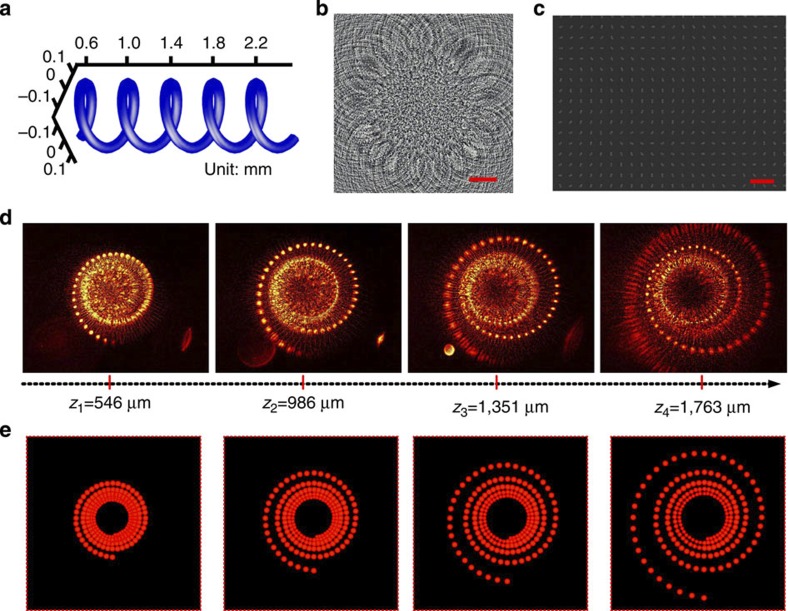
Holography of a 3D helix. (**a**) Geometry of the helix. (**b**) The calculated hologram and (**c**) scanning electron microscopy view of the constituent nanorods pattern. Scale bars, 20 μm and 1 μm in **b** and **c,** respectively. (**d**) On-axis evolution of the total five turn of the helix by tuning the focusing position along the *z* direction. (**e**) Numerical calculation of the 2D perspective view by taking into consideration the position-dependent magnification.

**Figure 6:**
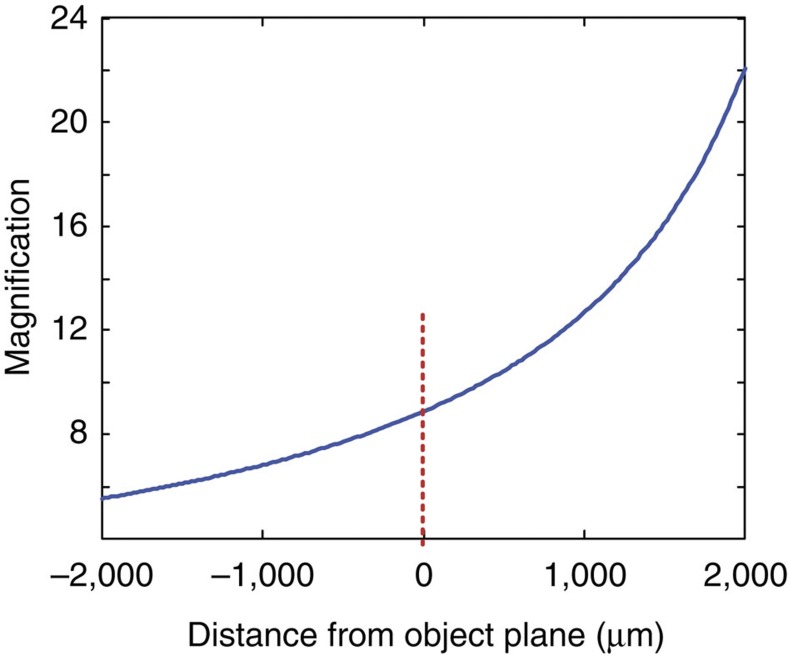
Position-dependent magnification of the optical measurement system. The magnification of our imaging system is obtained from a ray tracing calculation. It shows a nonlinear dependence on the distance of the holographic image to the object plane.

We further investigate the performance of 3D metasurface holography for a five-turn hollow helix pattern (400 μm pitch, 150 μm diameter), with the helix axis along the *z* direction (perpendicular to the metasurface). The target 3D object, the CGH image of the overall hologram pattern and the scanning electron microscopy image of its constituent dipole antennas are shown in Fig. 5a–c, respectively. The hollow helix is specifically designed for the demonstration of different perspective views of the 3D image. First, by tuning the object plane along the *z* direction, the on-axis evolution of the five-turn helical image is measured (Fig. 5d). For each 2D image slice, at least one complete helix pitch (400 μm along the *z* direction) can be seen clearly. The recording of 3D images on a 2D plane in general shows some signature of perspective, that is, the size of the image changes with distance to the observer. As the perspective of our imaging system is not linear, the magnification for the sections of helix that fall out of focus shows a nonlinear dependence on its *z* location. The distance-dependent magnification in our imaging system is obtained by a ray tracing calculation (see Fig. 6), which is subsequently used to reconstruct the perspective image on a 2D plane at the location of the CCD camera. The calculated 2D perspective images of the 3D helix, shown in Fig. 5e, show reasonable agreement with the observed images. Note that the calculated perspective images do not show the blurry out-of-focus effect, as the calculation does not take into account the finite depth of focus in the real imaging system.

**Figure 7:**
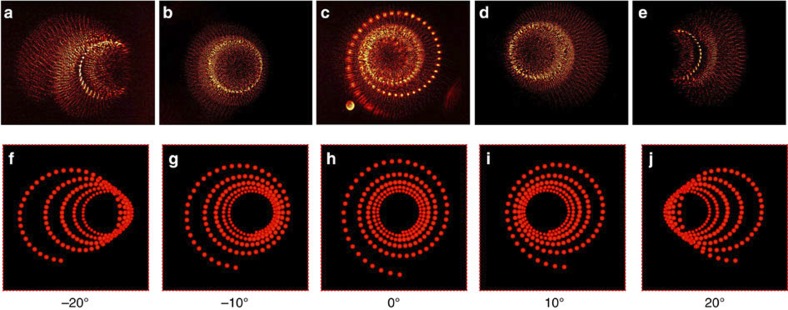
Holographic images at tilted observation angles but normal incidence illumination. (**a**–**e**) Holographic image observed at different angles with *z*=1,351 μm. (**f**–**j**) The corresponding ray tracing calculations of the 2D perspectives of the holographic images.

When rotating the imaging system while keeping the illuminating system and metasurface fixed, an oblique view of the 3D holographic image can be obtained. The holographic images for viewing angles ranging from −20° to 20° at steps of 10° are shown in Fig. 7a–e, which clearly show the tilting effect and further verify the 3D nature of the holographic image. Note that at larger viewing angles of ±20°, only part of the holographic image can be captured by the objective because of the finite beam divergence of the light forming the holographic image. Again, the experimental observations can be qualitatively explained by the 2D perspective images calculated by ray tracing shown in Fig. 7f–j. From our experiment, we estimate the FOV to be in the range of −40° to 40°.

## Discussion

As the 3D objects demonstrated here consist of diffuse reflecting surfaces or point sources, the amplitudes of the holograms are highly uniform, thus eliminating the need for amplitude control without sacrificing the image quality. On the other hand, if multiple reflection properties of the object surfaces are desired, such as specular reflection and phong shading, a complex amplitude modulation of the incident light can show better performance. Nevertheless, complex amplitude modulation may be obtained in the metasurface by additionally encoding the amplitude information into the length of the nanorods. This will lead to a modification of the resonance frequency and therefore different scattering amplitude.

Current holography technologies are mainly limited by the large pixel size of the holograms. Specifically, the FOV is determined by the maximum diffraction angle of the hologram, which is given by: sin *θ*=
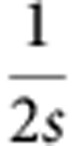
*λ*, where *s* is the pixel pitch of the hologram. For example, SLMs, despite being dynamic, have pixel sizes of at least 6.4 × 6.4 μm^2^, which are more than one order of magnitude larger than the wavelength of light. Therefore, for a SLM-based hologram, the FOV is far from being desired for 3D holography. Holograms based on diffractive optical element, superficial microrelief with depths on the order of optical wavelength, usually have pixel sizes of at least 10 wavelengths, such that the scalar diffraction theory can be applied^51^. Below this length scale, rigorous vector diffraction theory would be required which are computationally time consuming. In addition, the multiphase level in diffractive optical element necessary to produce high-quality holography entails multiple steps of lithography with precise alignment. Recently, researchers have reported amplitude-based holograms using carbon nanotubes^46^ with pixel sizes down to 400 × 400 nm^2^. However, the binary nature of the holograms results in significantly less information per pixel than the continuous phase profiles in our metasurface holograms, and thus compromising the quality of image reconstruction. In addition, the problems such as twin image and zero-order diffraction are present in such amplitude holograms. Bulk metamaterial-based holograms^42^ are so far only designed to work at infrared wavelengths because of the fact that they require complex fabrication processes involving multilayer alignment. Thus, compared with other methods for generating holograms, the metasurface approach presented here provides easy access to subwavelength pixel sizes with continuously controllable phase profiles. This advantage arises from the fact that the geometrical size of the plasmonic nanoantennas (here around 150 nm) is much smaller than the free-space wavelength used for generating the image (670–950 nm), and the capability of generating well-controlled continuous phase profile benefits from the simplicity in the encoding of phase information into the orientation of the plasmonic nanorods.

In summary, we have demonstrated on-axis plasmonic holography for distortion-free 3D images in the visible and near-infrared range by using subwavelength pixelated plasmonic metasurfaces. A complete phase control is achieved without an extra look-up table or phase accumulation along an optical path. The subwavelength pixel size of the metasurface hologram represents a great advantage over other conventional methods such as CGH with SLMs or diffraction optical elements and, more importantly, the common issue of multiple diffraction orders accompanying 3D holographic images is avoided. The dispersionless nature of our metasurface can result in broadband operation without sacrificing image quality. Such a scheme could potentially be used in high-resolution holographic data storage, optical information processing and other holography-based techniques.

## Methods

### Computer-generated hologram design

Point source algorithm and Fresnel diffraction theory are used for the generation of the CGH. In our phase-only hologram design, proper choice of random phase is added to the point sources constituting the 3D object to mimic the diffuse scattering body, which is in contrast to the specular holography. Thus, standard normal (Gaussian)-distributed pseudorandom phase *X*~2*π*·*N*(*μ*, *σ*^2^), with *μ*=0 and *σ*=1 (in the unit of radian) is added to each pixel of the target object to achieve a more uniform amplitude distribution.

Through angular spectrum method, we can numerically reconstruct the 3D objects to verify the design. In addition, to mimic the possible phase shifts due to fabrication errors, a certain range of random phase can be added to the hologram, and by angular spectrum method we have numerically verified that such hologram is very robust against phase noise.

## Author contributions

L.H., X.C., T.Z. and S.Z. proposed the idea, L.H., H.Z., T.Z. and S.Z. conducted pattern designs and numerical simulations, H.M. fabricated the samples, X.C., L.H. and S.C. performed the measurements, L.H., X.C., J.L., C.-W.Q., T.Z. and S.Z. prepared the manuscript. S.Z. and T.Z. supervised the overall projects. All the authors analysed the data and discussed the results.

## Additional information

**How to cite this article:** Huang, L. *et al.* Three-dimensional optical holography using a plasmonic metasurface. *Nat. Commun.* 4:2808 doi: 10.1038/ncomms3808 (2013).
